# Importance of SERCA2a on early isolated diastolic dysfunction induced by supravalvular aortic stenosis in rats

**DOI:** 10.1590/1414-431X20175742

**Published:** 2017-04-13

**Authors:** C.F.S.M.P. Silveira, D.H.S. Campos, P.P. Freire, A.F. Deus, K. Okoshi, C.R. Padovani, A.C. Cicogna

**Affiliations:** 1 Departamento de Clínica Médica, Faculdade de Medicina de Botucatu, Universidade Estadual Paulista, Botucatu, SP, Brasil; 2 Departamento de Bioestatística, Instituto de Biociências, Universidade Estadual Paulista, Botucatu, SP, Brasil

**Keywords:** Papillary muscle, Echocardiogram, Cyclopiazonic acid, Rat, Isolated diastolic dysfunction, SERCA

## Abstract

Cardiac remodeling is defined as changes in shape and function of the heart in response to aggression (pressure overload). The sarcoplasmic reticulum calcium ATPase cardiac isoform 2a (SERCA2a) is a known factor that influences function. A wide spectrum of studies report a decrease in SERCA2a in heart failure, but none evaluate it's the role in early isolated diastolic dysfunction in supravalvular aortic stenosis (AoS). Our hypothesis was that SERCA2a participates in such dysfunction. Thirty-day-old male Wistar rats (60-80 g) were divided into AoS and Sham groups, which were submitted to surgery with or without aorta clipping, respectively. After 6 weeks, the animals were submitted to echocardiogram and functional analysis by isolated papillary muscle (IPM) in basal condition, hypoxia, and SERCA2a blockage with cyclopiazonic acid at calcium concentrations of 0.5, 1.5, and 2.5 mM. Western-blot analyses were used for SERCA2a and phospholamban detection. Data analysis was carried out with Student's *t-*test and ANOVA. AoS enhanced left atrium and E and A wave ratio, with preserved ejection fraction. Basal condition in IPM showed similar increases in developed tension (DT) and resting tension (RT) in AoS, and hypoxia was similar between groups. After cyclopiazonic acid blockage, final DT was equally decreased and RT was similar between groups, but the speed of relaxation was decreased in the AoS group. Western-blot was uniform in all evaluations. The hypothesis was confirmed, since functional parameters regarding SERCA2a were changed in the AoS group.

## Introduction

Cardiac remodeling (CR) can be defined as changes in genetic expression, reflected in alterations in molecular, structural and functional characteristics, as a response to specific aggressions, such as ischemia, inflammation, genetic mutations and volumetric or pressure overload (1–3). CR is time-dependent and in the long term it may be a threat, constituting an important risk factor for the development of ventricular dysfunction and heart failure (HF) (4).

Sarcoplasmic reticulum calcium ATPase cardiac isoform 2a (SERCA2a) is among the several factors that control cardiac function (5). The activity of this protein, which works by recapturing calcium into the sarcoplasmic reticulum during diastole, is an important determinant to myocardial performance (6). A wide spectrum of studies in humans relating SERCA2a to systolic HF can be found in the literature, showing a decrease in both its transcription and translation (7–10). The possibility of this protein being connected to HF pathophysiology has inspired experimental studies involving transgenic animals. Louch et al. (11), using a SERCA2a knock-out mice model, reported increased diastolic and systolic dysfunction in these animals. On the other hand, some studies have found that SERCA2a overexpression was followed by an improvement in contractile and relaxation function in failing hearts (12–14). Regarding non-transgenic experimental models for HF, such as spontaneously hypertensive animals and dilated cardiomyopathy, the literature generally seems to agree about the decrease in SERCA2a at systolic dysfunction (15–17). There also seems to be a consensus that SERCA2a levels decrease in myocardial dysfunction with severe heart failure, which are cases with both systolic and diastolic impairment induced by aortic stenosis (AoS) (18). Despite this evidence, there is no available study evaluating the relationship between SERCA2a levels and heart function in hypertrophy models, specifically supravalvular AoS, with isolated diastolic dysfunction at an early stage diagnosed by echocardiogram.

The objective of this research was to evaluate the participation of SERCA2a in premature diastolic impairment, with improved or normal systolic function in hypertrophied hearts induced by supravalvular AoS.

## Material and Methods

### Animal care

Thirty-day-old male Wistar rats (60-80 g) obtained from the Animal Center of Faculdade de Medicina de Botucatu, UNESP (Botucatu, SP, Brazil) were housed in individual cages. The environment was controlled in terms of light (12-h light/dark cycle starting at 6:00 am) and clean-air room temperature (23°C). All experiments and procedures were performed in accordance with the Guide for the Care and Use of Laboratory Animals published by the National Research Council (1996) and were approved by the Faculdade de Medicina de Botucatu Ethics Committee (No. FMB-PE-69/2009).

### Experimental protocol

#### Aortic stenosis surgery and experimental groups

The supravalvular AoS model is well established in scientific literature as a left ventricle (LV) hypertrophy promoter (3,4,19–24). It is defined by an initial light overload followed by a slow progression, proportional to the animal's growth.

The rats were submitted to anesthesia with ketamine (50 mg/kg, *im*). A thoracotomy was performed and a silver band (0.6 mm of internal diameter) placed around the ascending aorta, as previously described (25), constituting the AoS group (n=32). For the control group, rats were submitted to the same surgery but without aortic banding (Sham, n=32). After 6 weeks, all animals were submitted to echocardiogram and subdivided into two different experiments, according to treatment: SERCA2a blockage (Sham, n=15; AoS, n=11) or hypoxia (Sham, n=16; AoS, n=18).

Exclusion criteria for this study were animals showing clinical or laboratory evidence of systolic dysfunction or heart failure.

#### In vivo functional and structural study

All rats were submitted to echocardiographic evaluation 6 weeks after surgery, since previous studies have shown a diastolic dysfunction and improved systolic function progression from 2 to 6 weeks (3). A commercially available echocardiograph (General Electric Medical Systems, Vivid S6, Israel) equipped with a 5-11.5 MHz multi-frequency probe, was employed as previously described (26–29). Rats were anesthetized by intramuscular injection of a mixture of ketamine (50 mg/kg) and xylazine (0.5 mg/kg). The following structural variables were measured: left atrium diameter (LA), LV diastolic and systolic diameters (LVDD and LVSD, respectively), LV diastolic posterior wall thickness (DPWT), relative wall thickness (RWT) and the aortic diameter (AO). Left ventricular function was assessed by the following parameters: heart rate (HR), midwall fractional shortening (FS), ejection fraction (EF), early and late diastolic mitral inflow velocities (E and A waves) and E/A ratio.

#### In vitro functional study

According to the protocol currently employed in our laboratory (30,31), under anesthesia and following a thoracotomy the hearts were quickly removed and placed in oxygenated Krebs-Henseleit solution at 28°C. The papillary muscle was dissected carefully from the left ventricle, clipped at its edges, placed vertically in a chamber containing Krebs-Henseleit solution at 28°C, oxygenated with a mixture of 0.95 O_2_ and 0.5 CO_2_, pH 7.38, and stimulated with two electrodes inside the solution at a rate of 0.2 Hz. The basal composition of Krebs-Henseleit solution in mM was as follows: 118.5 NaCl, 4.69 KCl, 2.5 CaCl_2_, 1.16 MgSO_4_, 5.50 glucose and 25.88 NaHCO_3_. After a 60-min period, during which preparations were allowed to shorten while carrying light loads, muscles were loaded to contract isometrically and stretched to the apices of their length-tension curves. After a 5-min period, during which preparations performed isotonic contractions, muscles were again placed under isometric conditions and the apex of the length-tension curve (L_max_) was determined.

The following basal parameters were measured from isometric contraction: peak developed tension (DT, g/mm^2^), resting tension (RT, g/mm^2^), time to peak tension (TPT, ms), maximum rate of tension development (+dT/dt, g·mm^−2^·s^−1^) and maximum rate of tension decline (−dT/dt, g·mm^−2^·s^−1^). The mechanical behavior of papillary muscle was evaluated in basal condition, after hypoxia and SERCA2a blockage by cyclopiazonic acid (CPA). Since SERCA2a is an ATPase and oxygen-dependent, we submitted the IPM of both groups to hypoxia, targeting to evaluate whether the group with AoS presented any oxygenation deficits (32), by bubbling the solution through with 0.50 O_2_, 0.45 N_2_ and 0.05 CO_2_. During reoxygenation, the original 0.95 O_2_ and 0.05 CO_2_ gas mixture was used. The SERCA2a blockage was performed using the highly specific blocker CPA (Penicillium cyclopium, Sigma¯-Aldrich, USA), 30 mM, in the presence of cumulative calcium concentrations (0.5, 1.5 and 2.5 mM). Sixty minutes after CPA addition to the solution, each concentration of calcium was separately added to the bathing solution, and the papillary muscle response was analyzed 10 min after the addition. DT, RT and -dt/DT are reported as percentage of recovery (%), in relation to each calcium concentration's value before CPA. CPA solution was prepared as described previously by Badaoui et al. (33), by mixing 5 mL of dimethyl sulfoxide (DMSO) to the 10 mg recipient of CPA and then adding 252 µL of such solution to each chamber already filled with 50 mL of Krebs-Henseleit solution.

Papillary muscle cross-sectional area (CSA) was calculated from muscle weight and length by assuming cylindrical uniformity and specific gravity of 1.0. All force data were normalized for muscle CSA. Papillary muscles with CSA >1.5 mm^2^ were excluded from analysis as they can present central core hypoxia and impaired functional performance.

#### Postmortem structural study

At the completion of the functional study, the right and left ventricles (including the interventricular septum) were dissected, separated and weighed. Measurements of right and left ventricle and atria weight, normalized by final body weight, were used as hypertrophy indexes. Lungs and liver wet weight/dry weight were employed to determine whether there was an increase in body water.

#### Molecular study

The myocardial SERCA2a levels in both groups were evaluated by protein expression. As the function of SERCA2a is modulated by its physical interaction with the phosphoprotein phospholamban (PLB), which inhibits SERCA2a activity in its unphosphorylated state, PLB protein expression and the SERCA2a/PLB ratio were also determined. These proteins were assessed by western blot analysis according to the currently accepted procedure.

#### Histological analysis of total collagen in left ventricle

Samples of the left ventricle were put in a 10% solution of formaldehyde in water over 24 h. Next, the tissue was washed in running water for 24 h and then transferred to an ethanol 70% solution and included in a paraffin block. Five-micrometer histological slices were stained with Picrosirius red. We analyzed 9 Sham and 8 AoS animals, using an average of 30-40 microscopic fields per slide, and the myocardial fibrosis analysis was performed through interstitial collagen quantification. The histological slices were projected in a 40-time increase with a LEICA microscope (Leica Microsystems CMS, Germany), connected with a video camera, which sent the digital images to a computer with an image analysis software (Image Pro-plus, Media Cybernetics, USA). The components of the tissue were identified according to its color: red meaning collagen fibers; yellow, myocytes and white, interstitial space.

### Statistical analysis

Data from general characteristics, cardiac function and molecular analysis are reported as means±SD. Comparisons between groups were performed using Student's *t*-test for independent samples. Data from the hypoxia experiment are reported as means±SD and submitted to the multivariate analysis for repeated measures model in independent groups, complemented with the Bonferroni's multiple comparison test. Collagen data were analyzed using Mann-Whitney test for non-parametric measures. SERCA2a blockage maneuver is reported as median, maximum (max) and minimum (min) value, and studied using analysis of variance for repeated measures, complemented with the multiple comparison test of Student-Newman-Keuls, with a level of significance of 0.05.

## Results

### Postmortem morphological study

Table 1 shows the morphological analysis performed after euthanasia of both groups. AoS promoted a greater LV, right ventricle (RV) and atria (AT) weight, while it preserved final body weight. Moreover, there was no statistically significant difference between groups as to wet and dry weight of lungs and liver, which proves that those animals did not have heart failure, one of the exclusion criteria for this study.

**Table 1:**
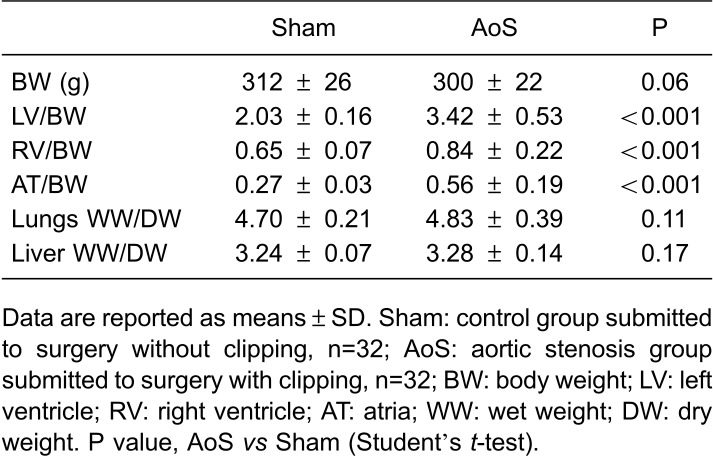
Postmortem structural study.

### *In vivo* functional and structural study

Table 2 summarizes the echocardiographic evaluation of both groups. There was statistical difference in structural data: LV relative wall thickness and LA/AO were significantly greater in the AoS group. There was no change in either EF or midwall FS (%), showing preserved systolic function. However, there was a difference in E wave and E/A, which were greater in AoS.

**Table 2:**
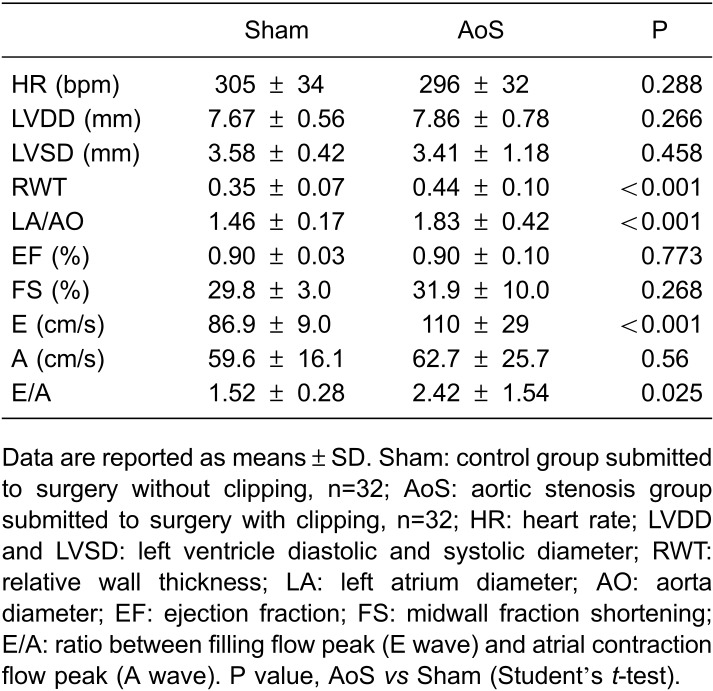
Functional and structural study results of echocardiogram.

### *In vitro* functional study

The evaluation of *in vitro* myocardial function using isolated papillary muscle in basal condition is represented in Table 3. The 6-week exposure to supravalvular AoS provoked a significant increase in RT and TPT, when compared to control group, while +dT/dt was decreased in the AoS group.

**Table 3:**
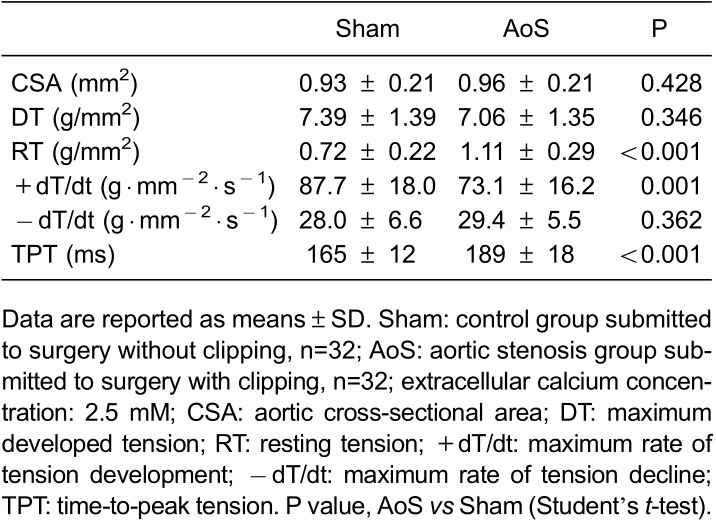
Basal isometric contraction.

**Figure 1:**
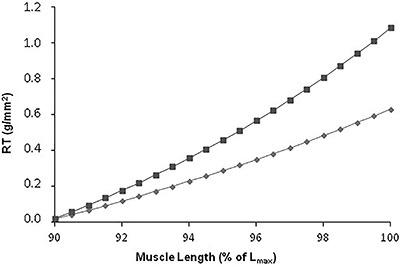
Relationship between muscle length and resting tension (RT) of aortic stenosis (AoS, dark grey, n=32) and control groups (Sham, light grey, n=32), using the progression model adjusted according to groups. Data are reported as mean±SD. P<0.05 (non-linear regression model adjustment using the minimum square method).

The influence of muscular length variation over RT is reported in Figure 1. There was a significant increase in RT in AoS compared to Sham rats.

**Figure 2:**
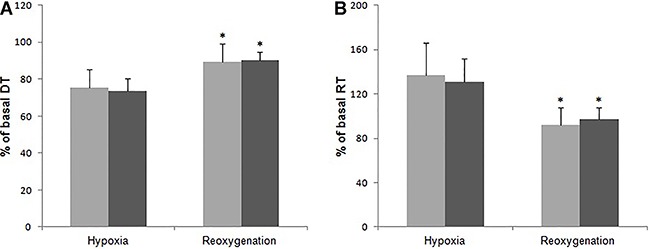
Influence of hypoxia (60 min) and reoxygenation (30 min) on (*A*) developed tension (DT - g/mm^2^) and (*B*) resting tension (RT - g/mm^2^) of isolated papillary muscle from aortic stenosis (AoS, n=18; dark gray columns) and control groups (Sham, n=16; light gray columns). Data are reported as means±SD. *P<0.001, *vs* hypoxia (multivariate analysis for repeated measures model in independent groups, complemented with Bonferroni's multiple comparison test).

The effects of hypoxia over papillary muscle, in relative values (%), regarding basal condition, are reported in Figure 2A and B. Sham and AoS groups achieved 75.3±5.4 and 73.7±6.6% of their respective basal condition DT, P>0.05. RT was increased to 137±29 and 131±21% of Sham and AoS basal condition, respectively, P>0.05. There was also no difference between groups after reoxygenation, for both DT (Sham: 89.2±4.6%; AoS: 90.4±4.2%, P>0.05) and RT (Sham: 91.7±16.1; AoS: 97.0±11.0%, P>0.05). However, there was a significant difference in both parameters at reoxygenation with respect to hypoxia within each group (P<0.001).

The effects of the SERCA2a blockage with CPA over DT, RT and -dT/dt are reported in Table 4, in percentage of basal conditions in each calcium concentration, in median, min and max. Both groups suffered a significant decrease on DT after adding CPA, reaching a maximum of 83% of basal condition DT (AoS, 2.5 mM Ca), thus demonstrating the efficacy of CPA blockage, which naturally will diminish contractile function due to its SERCA2a blockage mechanism. DT was different between groups AoS and Sham in all moments analyzed, maintaining the difference found in basal conditions, where AoS had greater systolic function, and also within groups between 2.5 and 0.5 mM of calcium, showing the importance of calcium concentration in contraction. As to RT, there was no difference between groups AoS and Sham in any moment analyzed. However, RT was greater within the AoS group at 1.5 and 2.5 compared to 0.5 mM of calcium, P<0.05. Also, at 2.5 mM calcium concentration, the AoS group presented a lower relaxation speed, described as -dT/dt compared to Sham, another indicator of diastolic dysfunction.

**Table 4:**
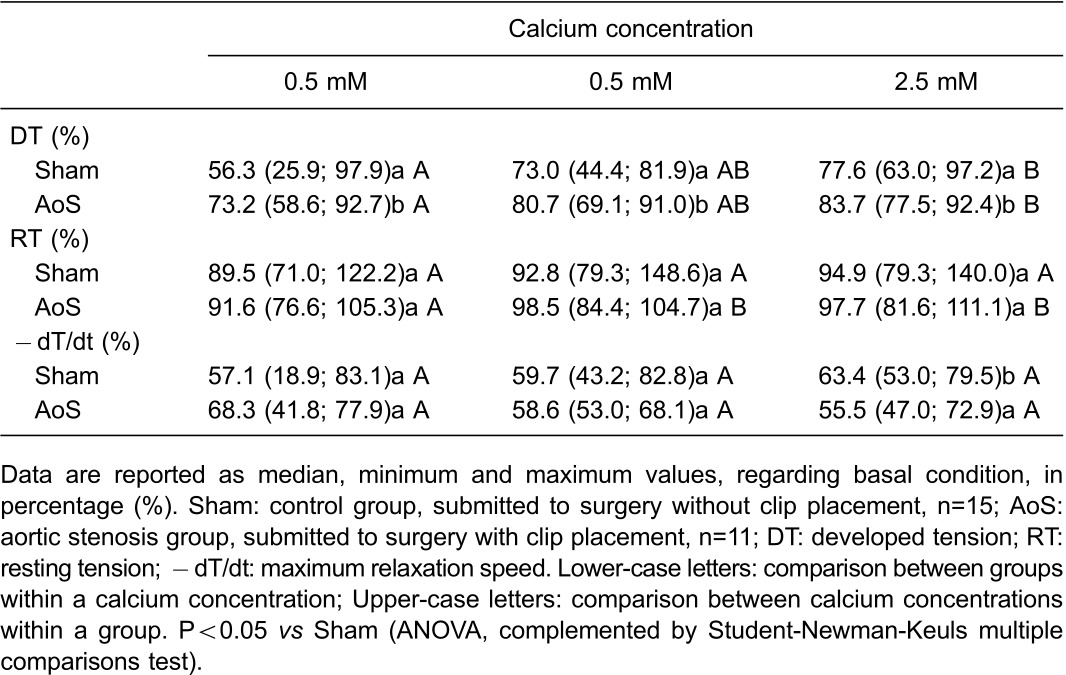
Cyclopiazonic experiment.

### Molecular study

**Figure 3:**
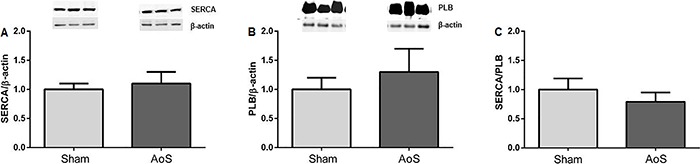
Protein expression of sarcoplasmic reticulum calcium ATPase (SERCA) (*A*) and phospholamban (PLB) (*B*) and relationship between SERCA and PLB (*C*), from aortic stenosis (AoS, n=6) and control group (Sham, n=6). Data are reported as means±SD. P values were 0.32, 0.12, and 0.16, respectively, *vs* Sham (Student’s *t*-test).

Data regarding western blot results are shown in Figure 3A-C. Quantified SERCA2a was similar between groups (Sham=1.00±0.18; AoS=1.10±0.19; P=0.32), as well as PLB (Sham=1.00±0.23; AoS=1.30±0.37; P=0.12) and SERCA2a/PLB ratio (Sham=1.03±0.20; AoS=0.87±0.17; P=0.16).

### Histological analysis of total collagen in left ventricle

**Figure 4:**
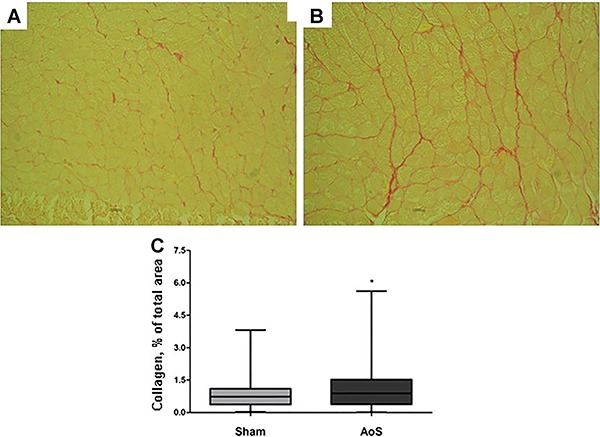
Histological image with Picrosirius red stain (40×). *A*, Sham group; *B*, aortic stenosis (AoS) group. *C*, collagen level, % of total area analyzed with Picrosirius red technique from AoS (n= 8) and control groups (Sham, n=9). Data are reported as median, maximum and minimum. *P<0.05 *vs* Sham (Mann-Whitney’s test).

Data concerning Picrosirius red staining are shown in Figure 4C. Analysis showed a difference between groups (P=0.001), based on the percentage of total collagen. Figure 4A and B illustrates the procedure and shows the difference found: myocytes were increased in size, showing the hypertrophy described previously, and the red color was much stronger in the AoS group.

## Discussion

The main goal of this study was to determine whether SERCA2a played a role in early isolated diastolic dysfunction assessed by echocardiogram in left ventricular hypertrophy caused by supravalvular AoS, by employing protein blocking during the isolated papillary study complemented by a molecular analysis. The results showed that such model was adequate for its purposes as it led, after 6 weeks, to an increase in the left atrium, E/A relation and preservation of ejection fraction. The diastolic dysfunction was then successfully reproduced in isolated papillary muscle, where there was an increased RT in basal conditions, as well as during muscle stretching, which is basically what was expected, since RT is the *in vitro* equivalent of diastolic function. Our functional studies showed that SERCA2a does participate in the diastolic dysfunction provoked by AoS.

The echocardiogram showed that the AoS group had wider left atrium, thicker muscle wall and greater relative wall thickness in the left ventricle - compatible with concentric hypertrophy -, preserved ejection and midwall fraction shortening and a higher E/A relation, consistent with isolated diastolic dysfunction. As A wave was similar between groups, such difference was due to E wave, which was increased in AoS. These data lead us to classify these hearts in early diastolic dysfunction, because the A wave had not yet fallen. The majority of previous studies in our laboratory had not found such an increase in E/A relation (20 [Bibr B21] [Bibr B22] [Bibr B23],^24^,^25^).

Post-mortem analysis confirmed the morphologic echocardiogram data, as it showed heavier left ventricle, atria and right ventricle in the AoS group, consistent with previous studies (19). This information confirms cardiac hypertrophy, but puts in question whether there was also a right-side insufficiency. The similarity in lung and liver wet weight over dry weight between groups clarified that question and corroborated the isolated left diastolic dysfunction without heart failure.

When transporting the functional study to an *in vitro* environment, with isolated papillary muscle, basal condition showed preserved DT, diminished +dT/dt and increased TPT. What can be stated from that is that the AoS muscle had, as previously seen in echocardiogram, preserved systolic function. However, despite similar DT between groups, the alterations in both TPT and +dT/dt may show a decreased quality of contraction, even with an acceptable end result, which may indicate a change in myosin pattern with increased expression of its heavy chain slow isoform (34). As to RT, it was increased in the AoS group in basal conditions and during muscle stretching, where there was an important difference in the AoS group behavior, showing greater myocardial rigidity in comparison with the control group. These findings could be related to remaining calcium bound to C troponin secondary to an increase in end-diastole cytosolic calcium, along with excess of other proteins, such as titin, the components of extracellular matrix, cytoskeleton and structural alterations, due to the addition of sarcomeres in parallel that may also diminish ventricular compliance in AoS (35).

Therefore, we performed an analysis of the collagen to see whether it might be a component of the inherent increased RT found in AoS, although it would not explain the positive effects of CPA blockage found in that group. Using the Picrosirius red technique, the AoS group was found to have a much larger ratio of collagen/total area.

According to the objective of this study, we intended to analyze the participation of SERCA2a in the early isolated diastolic dysfunction found, through specific blockage of this protein in isolated papillary muscle. However, as SERCA2a is an ATPase, we first had to exclude the possibility of hypoxia interference in the maneuver. Thus, we submitted both groups to a relative hypoxia and analyzed their behavior. Both groups equally improved RT after reoxygenation, and there were no differences between groups regarding this variable during hypoxia. These data show that both groups suffered equally from hypoxia during the experiment, allowing to conclude that the results from SERCA2a blockage would be only due to its activity/number and not to oxygen supply.

The specific SERCA2a blockage with cyclopiazonic acid proved itself effective since it provoked a decrease in systolic function - here represented by DT - in both groups, although with a smaller decrease in the AoS group, probably due to a greater amount of myofilaments available in that group. Such decrease in DT is expected due to the SERCA2a blockage mechanism of CPA, which will impair the calcium transients. The fact the there was no difference between groups as to that variable just reinforces the distinct contractile function found on basal condition. The percentage of recovered RT in relation to each group's basal values was equal between the groups Sham and AoS, at all calcium concentrations. Such finding would at first lead us to believe that the CPA experiment did not have an effect over diastolic function, as SERCA2a blockage would mainly interfere with calcium recapture, thus causing a disruption in myosin-actin detachment. However, the AoS group presented a significant increase in recovered RT at 1.5 and 2.5 mM regarding 0.5 mM calcium concentration, which did not happen in the control group. Also, the speed of relaxation after blockage given by -dT/dt was slower in the AoS animals at 2.5 mM of calcium concentration, which indicates a deficient calcium transient. Although there was no difference between groups for RT, which may have occurred due to the great variability found among Sham animals, the fact that the AoS group presented a distinct behavior from control, with greater RT as calcium concentration increased associated with the slower relaxation found in that group allowed us to infer that the AoS group presented some sort of calcium recapture dysfunction related to SERCA2a. To conclude this protein's analysis, a molecular study was performed, through measuring SERCA2a and its regulator, PLB. Neither of them were increased or decreased in the AoS group, despite evidence of the contrary in the literature, which has found a decrease in both SERCA2a and PLB, not specifying the ratio findings in severe heart dysfunction (4,36,37).

The molecular findings go against the functional study with SERCA2a blockage. Although the present study did not show such decrease in SERCA2a, it is likely that our findings are related to a decreased activity causing recapture speed deficit, as well as to a mismatching relation between this protein and myofibrils content - which are increased in the hypertrophied muscle, only revealed after SERCA2a specific blockage. Summarizing, the results show that the hypertrophied muscle found in the early stage of AoS apparently presented inappropriate SERCA2a activity, which began to jeopardize the relaxation phase of the muscle.

In conclusion, the hypothesis of this study was confirmed, since functional parameters considering the *in vitro* environment regarding SERCA2a protein were altered in the AoS group, leading us to deduce that this protein must participate in early isolated diastolic dysfunction caused by supravalvular AoS. By being reproducible in *in vivo* hearts, this finding opens doors to the next step in isolated diastolic dysfunction research: defining targets on which drugs might act in order to stop or prevent the natural evolution of the disease. However, other contributing factors to this condition cannot be excluded for now and should be studied further.
